# Constant herbivory rates and plant–herbivore interactions along a resource availability gradient in South African fynbos

**DOI:** 10.1007/s00442-025-05793-2

**Published:** 2025-10-08

**Authors:** Alexander Neu, Huw Cooksley, Karen J. Esler, Anton Pauw, Francois Roets, Frank M. Schurr, Matthias Schleuning

**Affiliations:** 1 Senckenberg Biodiversity and Climate Research Centre (SBiK-F), Senckenberganlage 25, 60325 Frankfurt am Main, Germany; 2 Institute for Ecology, Evolution and Diversity, Goethe University Frankfurt, Frankfurt am Main, Germany; 3 Institute of Landscape and Plant Ecology, University of Hohenheim, Stuttgart, Germany; 4 Department of Conservation Ecology and Entomology, Stellenbosch University, Stellenbosch, South Africa; 5 Plant Conservation Unit, Department of Biological Sciences, University of Cape Town, Cape Town, South Africa; 6 Center for Biodiversity and Integrative Taxonomy (KomBioTa), University of Hohenheim, Stuttgart, Germany

**Keywords:** Ecological networks, Endophagous insects, Plant–animal interactions, *Protea*, Seed predation

## Abstract

Plant–herbivore interactions are important for almost all terrestrial ecosystems, but little is known about how herbivory and the specialization of these interactions change with the resource availability provided by host plant communities and time since disturbance. In fire-prone scrublands of the South African Cape Floristic Region, we studied interaction networks between 20 *Protea* shrub species and ten herbivorous insect species that consume seeds inside *Protea* cones during their larval stage. We studied these interactions at 18 sites that differed widely in plant resource availability (protea cone mass per hectare) and time since the last fire event. We sampled 1173 protea cones, identified the herbivores in each cone, and calculated herbivory rate (the proportion of infested cones), herbivore diversity (herbivore species richness and Shannon diversity), the number of host plant species per herbivore species (insect generality), the number of herbivore species per host plant species (plant vulnerability), as well as niche overlap among insect and plant species for each site. We found that most herbivore species interacted with the majority of *Protea* species. Herbivory rate and herbivore diversity were not affected by site-level resource availability or time since fire. Surprisingly, specialization of plant–herbivore interactions at the community level was independent of the environmental gradients studied, suggesting that the mechanisms structuring the interactions in this plant–herbivore system were independent of the environmental context. This finding suggests coupled community dynamics of protea plants and the insect herbivores feeding inside their cones in South African fynbos ecosystems.

**Supplementary Information:**

The online version contains supplementary material available at 10.1007/s00442-025-05793-2.

## Introduction

One important driver of ecological communities is the interactions between co-occurring species. Many of these interactions are beneficial for one partner and negative for the other, and these antagonistic interactions play a key role in community dynamics (Chesson and Kuang 2008; Bascompte 2010). The highly diverse antagonistic interactions between plants and herbivorous insects are relevant to almost all terrestrial ecological communities, with the host specificity of herbivorous insects varying at small and large spatial scales (Lewinsohn et al. 2005). On a global scale, herbivorous insects become more specialized with decreasing latitude, in line with the higher abundance of herbivorous insects and a higher diversity of host plant species in tropical regions (Dyer et al. 2007; Forister et al. 2015). At the community scale, the host specificity of herbivorous insects can span the entire range from generalism to specialism (Sudta et al. 2022), although specialized herbivores may be more common and occupy smaller home ranges than generalist species at this scale (Novotný et al. 2010). However, a few studies have tested how herbivore diversity, and especially herbivore specialization, may depend on the availability of plant-based resources at the community scale.

Herbivorous insects depend entirely on resources provided by plants (Novotný et al. 2006; Lewinsohn and Roslin 2008; Forister et al. 2015). Consequently, the abundance and diversity of herbivorous insects are often regulated by plant resource quantity and quality (Carmona et al. 2011; Ali and Agrawal 2012). The resource concentration hypothesis posits that herbivorous insects select areas for feeding and reproduction where their host species are abundant, and accordingly, where the availability of plant resources is high (Root 1973). However, support for this hypothesis varies as herbivorous insect population dynamics are often inconsistent (Marques et al. 2000; Otway et al. 2005). While several studies found increased herbivory with increased availability of plant resources (Sholes 2008; Barbosa et al. 2009; Andersson et al. 2013a; Calixto et al. 2023), some showed no relationship (Grez and González 1995), and others revealed an opposite ‘resource dilution’ pattern (Le Maitre 1984; Elzinga et al. 2005; Knight et al. 2013). It is unclear why herbivorous insect species exhibit resource concentration or resource dilution patterns, but the observed patterns may be related to how herbivores identify their hosts or how they feed on them (Prokopy and Owens 1983; Andersson et al. 2013b). Differences in plant species abundance among sites can also trigger changes in herbivores’ host plant preferences, affecting both specialized and generalized herbivorous insect species (Nobre et al. 2016).

In addition to the availability of plant resources, herbivore–plant interactions can be influenced by interactions between herbivores, such as competition for limited plant resources (Finke and Snyder 2008). For example, increased competition among herbivores feeding on the same host plant species (Kaplan and Denno 2007) can lead to competitive exclusion of some herbivorous species (Anderson et al. 2009). Closely related and co-occurring generalist herbivorous insects may indirectly compete for resources on the same plant taxon, but may differ in their feeding strategies (Behmer and Joern 2008). In addition to host plant diversity, niche partitioning among herbivorous insects is therefore a major driver of their diversity (Albrecht et al. 2023).

Herbivory can have severe effects on plant population dynamics and the diversity of plant communities (Maron and Crone 2006; Bagchi et al. 2014). This is especially apparent for predation of seeds before dispersal, which strongly affects the fitness of both host plants and herbivores (Nottebrock et al. 2013; Walter et al. 2022). As a consequence, plants have developed diverse defense strategies to deter insect herbivory (Wright 1994; Kessler and Baldwin 2002). This has resulted in a co-diversification between plant species and herbivorous insects (Janz 2011; Albrecht et al. 2023). The diversity of defense strategies against herbivores displayed among plant taxa is therefore an important driver of herbivore specialization on specific host plants (Ehrlich and Raven 1964; Forister et al. 2015).

Plant–herbivore interactions have been widely studied and compared across large spatial extents (Lewinsohn et al. 2005), e.g., along latitudinal (Dyer et al. 2007; Forister et al. 2015), elevational (Pellissier et al. 2018), or land-use gradients (Neff et al. 2021), as well as in communities varying in plant species richness (Unsicker et al. 2006; Andrew et al. 2012). However, a few studies have explicitly tested how changes in resource availability (i.e., the availability of plant-based resources for herbivores at a site) affect plant–herbivore interactions, even though the availability of plant resources is crucial for plant–herbivore interactions (Price 2002; Shin et al. 2021). Additionally, most studies testing the effect of resource availability on herbivorous insects used proxies, e.g., plant density or canopy cover (Shin et al. 2021), but did not quantify the actual amount of resources available to herbivores.

The frequency of disturbances in ecological communities is another important factor shaping plant–animal interactions (Dayton 1971; Pickett and White 1985). Fire is a disturbance that leads to sudden and idiosyncratic changes in the resource and habitat landscape for herbivores, such as the elimination of plant biomass and an abrupt decrease in resource availability (Dafni et al. 2012). Fire can have direct impacts on populations of herbivorous insects, with the magnitude depending on fire intensity and insect mobility (Swengel 2001; Bieber et al. 2023). After a fire, recolonization by herbivores depends on the number of local survivors and immigration from unburned areas around the burned area (Panzer 2003). Plants may benefit from such fire-induced disruption of their antagonistic interactions with specialized seed predators (García et al. 2016), but studies examining the effects of fire-triggered succession of plant communities on plant–herbivore interactions are rare (Le Maitre 1984; Dole et al. 2023).

An ideal study system to investigate the interactions between plants and herbivorous insects is the genus *Protea* (Proteaceae) of the nutrient-poor and fire-prone Cape Floristic Region (CFR, Goldblatt 1978). Proteas have specialized root structures and physiology to maximize phosphorus acquisition from soils (Lambers et al. 2006) and invest a significant amount of phosphorus into their canopy seedbanks (Groom and Lamont 2010). Therefore, seeds in Protea infructescences (hereafter cones) hold considerable amounts of essential nutrients (Low and Lamont 1986; Groom and Lamont 2010) and are a major source of phosphorus for herbivorous insects that have specialized in feeding on these plant resources in the CFR (Hunter 2010; Lambers et al. 2015). All CFR proteas are serotinous, that is, they store their seeds in fireproof cones (Bond 1985). The storage time depends on the species and the amount of serotiny, but usually lasts several years, making them an ideal breeding location for endophagous insect larvae (Nottebrock et al. 2017b). Fire events kill most *Protea* species (with the exception of a few resprouting species), including the herbivorous insect larvae living within their cones (Carlson and Holsinger 2010). CFR protea communities are therefore well suited to study how disturbance and the resources provided by plants affect the diversity and specialization of plant–herbivore interactions (Kraaij and van Wilgen 2014; Bosc and Pauw 2020).

We tested how herbivorous insect abundance, diversity, and the specialization of plant–herbivore interactions changed along gradients of host plant resource availability and time since the last fire. We hypothesized that (1) herbivory and herbivore species richness in protea cones increase with increasing resource availability (Root 1973; Östergård and Ehrlén 2005) and time since the last fire (Bosc and Pauw 2020), which would expose host plants to increasing herbivore pressure. We further hypothesized that (2) herbivorous insects are limited in their host ranges by interspecific competition at low resource availability, whereas specialized herbivores immigrate to disturbed sites later than generalized herbivores (Bosc and Pauw 2020).

## Materials and methods

### Study sites and site-level disturbance

We chose 18 study sites situated within the Fynbos Biome (Goldblatt 1978) in the Western Cape, South Africa. The study sites of 120 m × 120 m and one of 200 m × 200 m (8530 m mean distance to each other) were chosen to contain several overstorey *Protea* species (2–7 species) and a wide range of individuals (42–116,493 individuals). We used the time after a fire event as a measure of site-level disturbance. We collected information on the last fire at each study site by interviewing landowners and evaluating the CapeNature Fire Database (http://bgis.sanbi.org/Projects/Detail/168) and freely available satellite imagery (NASA FIRMS application and NASA Worldview application). We confirmed these data by aging *Protea* plants on the study plots (Bond 1985; Treurnicht et al. 2016). The resulting disturbance gradient (time since fire) ranged from 6 to 25 years (Table S1). We conducted the fieldwork under the CapeNature permits 0028-AAA008-00262 and 0056-AAA008-00070.

#### Sampling of herbivorous insects

We harvested protea cones in 2017 and 2018 during the southern hemisphere winter and spring, from April to September, when both the abundance and richness of herbivorous insects are the highest (Rebelo 2001; Roets et al. 2006). We sampled the endophagous insect larvae feeding within the plant tissue by collecting protea cones from ideally five plant individuals per species and site. On each plant individual, we aimed to select cones from the three previous flower cohorts. Every cohort represents a flowering year, so our sampling allowed us to follow plant–herbivore interactions across 3 years (Cooksley et al. 2023). For example, we harvested the three cohorts of cones at site “held_2” 6 years after a fire event, which implies that the cones were exposed to herbivory when the fire event occurred 3, 4, and 5 years ago. In the further analysis, we then merged the three cohorts to obtain an average value for the 3 years, which helps to control for inter-annual fluctuations of herbivore pressure.

For each cohort, we randomly collected one open cone (seeds released due to abiotic influences such as drought stress or due to damage from herbivorous insect predation) and one closed (i.e., externally intact) cone. This resulted in a maximum of six cones collected per individual and a maximum of 30 cones per species and site. As the larvae of the different herbivorous insect species leave characteristic feeding marks in *Protea* cones (Neu et al. 2023), we were able to assign almost all traces found in closed and open cones to a specific herbivorous insect species. This was also possible if the insect had already left the cone. All herbivorous insect larvae and their larval cases found in the cones were stored in ethanol in a reference collection at Stellenbosch University.

#### Sampling of plant-based resources

For each site, we estimated the total availability of plant resources for the studied herbivorous insect larvae, as these insect species feed almost exclusively on proteas (Sasa and Samways 2015). The larval stages of endophagous herbivorous insects in protea cones primarily feed on protea seeds, but also on other parts of the cone, such as the receptacle (Coetzee and Gilomee 1987b). Hence, we used the dry mass of the entire cone as an integrative measure of host plant resource availability for the herbivores studied here. We restricted the estimated host plant resource availability to those provided by the cones of overstorey protea plants taller than 0.3 m, since they account for the vast majority of biomass in fynbos (van Wilgen 1982). To obtain site-level resource availability, we used individual-based mapping data from 17 study sites, initially implemented by Nottebrock et al. (2017a) and Schmid et al. (2015). We updated their mapping data where necessary and included one new site that was mapped in 2017 and 2018. Mapping involved recording the location of every protea individual with a differential GPS (Trimble GeoXH) as well as its species identity and height. To predict the number of cones for the three sampled cohorts per site, we first predicted the size of all protea individuals in the years of cone production, and from this, predicted the cone numbers produced on each plant in each year. We assumed a power-law relationship between plant size (height in the main growing axis) and age, a cubic neighbor index (NI) and a binary variable of plant sprouting (three of the studied protea species are resprouters, the rest non-sprouting, see Walter et al. 2022)

$${\text{E}}\left( {{\text{ln}}\left( {{\text{size}}} \right)} \right)\, = \,{\text{ln}}\left( {{\text{age}}} \right)\, + \,{\text{ln}}\left( {{\text{NI}}} \right)\, + \,{\text{sprouter}}\, + \,{\text{ln}}\left( {{\text{age}}} \right):{\text{ ln}}\left( {{\text{NI}}} \right)\, + \,{\text{ln}}\left( {{\text{age}}} \right):{\text{ sprouter}}\, + \,\left( {{\text{ln}}\left( {{\text{age}}} \right)|{\text{species}}} \right)\, + \,\left( {{1}|{\text{site}}} \right).$$

NI describes the influence of the J neighbors on the cone production of plant i, decreasing with the distance between them, d_i,j_

$$NI_{i} = { }\mathop \sum \limits_{j = 1}^{J} e^{{( - d_{i,j}^{3} )}} .$$

We fitted this model to our mapping data comprising more than 388,539 protea individuals, assuming a normal error distribution. Note that a cubic index produced the best fit out of inverse, exponential, Gaussian, and cubic indices, as assessed by the lowest Akaike Information Criterion and the highest coefficient of determination (conditional *r*^2^ = 0.76 in the best-fit model). We next fitted an analogous model to predict cone number from plant size

$${\text{E}}\left( {{\text{ln}}\left( {\text{cone number}} \right)} \right)\, = \,{\text{ln}}\left( {{\text{size}}} \right)\, + \,{\text{ln}}\left( {{\text{NI}}} \right)\, + \,{\text{sprouter}}\, + \,{\text{ln}}\left( {{\text{size}}} \right):{\text{ ln}}\left( {{\text{NI}}} \right)\, + \,{\text{ln}}\left( {{\text{size}}} \right):{\text{ sprouter}}\, + \,\left( {{\text{ln}}\left( {{\text{size}}} \right)|{\text{species}}} \right)\, + \,\left( {{1}|{\text{site}}} \right),$$

assuming a Poisson error distribution for cone number. This model was fitted to yearly stratified counts of cone production and size measures of the same focal individuals from which cones were sampled for insect identification (*n* = 672 protea individuals, conditional *R*^2^ = 0.69, Walter et al. 2022). Based on these models, we were able to estimate the cone number produced on every plant on our sites, for each of the three years before sampling. To remove outliers, we truncated the predictions of cone numbers to below the 95th percentile for each species and year.

To obtain measures of site-level cone dry mass for every species, we processed 1915 closed cones from non-focal plants (min. 1, max. 94 cones per species and site). We oven-dried the cones at 70 °C and measured their mass using a precision balance (accurate to 0.001 g). To obtain the site-level resource availability for the herbivore species studied here, we averaged the predicted cone numbers per site and species over the 3 years and multiplied this number by the population-level cone dry masses for each species at each site. Finally, we summed the species-specific values for each site. Across all study sites, the site-level resource availability ranged from 4990 to 2,115,268 g/ha (mean = 472,118 g/ha, Table S1). Resource availability was log-transformed prior to further analyses. Time since fire and site-level resource availability were not correlated (df = 16, cor = −0.335, *P* = 0.174). The number of *Protea* species per site was neither correlated with site-level resource availability (df = 16, cor = 0.322, *P* = 0.192) nor with time since fire (df = 16, cor = −0.363, *P* = 0.138).

### Statistical analysis

All metrics of herbivory, herbivore diversity, and specialization were controlled with a rarefaction analysis (Gotelli and Colwell 2001). To this end, we resampled cone data to the minimum number of cones sampled per site (*n* = 36 cones, without replacement) and calculated all metrics based on this sample for 100 bootstraps (as described below). We used the mean rarefied metrics across the 100 bootstraps for further analyses.

To address hypothesis 1 (herbivory and herbivore diversity increase with increasing resource availability and time since fire), we computed measures of herbivorous insect abundance and diversity for each site. To this end, we calculated the number of cones attacked by herbivorous insects per site divided by the number of sampled cones per site (*n* = 36), as a measure of overall site-level herbivory. We further calculated the number of herbivorous insect species (hereafter herbivore richness) per site and the Shannon diversity of herbivores at each site based on their relative proportions.

To address hypothesis 2 (herbivorous insects are more specialized at low resource availability and specialized herbivores immigrate later than generalized herbivores), we built site-level networks between *Protea* species and herbivorous insect species and counted the number of interactions between each co-occurring species pair. The co-occurrence of plant and herbivorous insect species was defined by the presence of a plant and an herbivorous insect species at each site. Based on these plant–herbivore networks, we calculated insect generality and insect niche overlap as measures of interaction diversity and niche overlap of insect species. Insect generality is defined as the effective number of plant species per herbivore species (Tylianakis et al. 2007; Dormann et al. 2009). We standardized generality by the number of sampled *Protea* species per site, so that it ranged between 0 and 1. Insect niche overlap quantifies the average similarity between herbivore species with respect to their host plants (Dormann et al. 2009). It was calculated using the Horn-Morisita index and ranged from 0 to 1, with 0 indicating no niche overlap and 1 indicating complete niche overlap (Krebs 1989). We further calculated plant vulnerability (Tylianakis et al. 2007) and plant niche overlap as measures of interaction diversity and niche overlap of *Protea* species from the same networks. Analogous to the insect-based measures, plant vulnerability is the mean number of herbivore species per host plant species, and niche overlap is based on the similarity of insect herbivores between *Protea* species. Plant vulnerability was standardized by the number of herbivore species recorded at each site. For each site, we calculated mean values of insect generality, plant vulnerability, and plant and insect niche overlap across all *Protea* species occurring at a site.

All network metrics were calculated with the “grouplevel” function from the “bipartite” package (version 2.16; Dormann 2011). We used linear models to test the relationships between the response variables (corresponding to each of our two hypotheses) and site-level resource availability and time since fire (*n* = 18 study sites). All analyses were performed using R version 4.1.0 (R Core Team 2021).

## Results

**Fig. 1:**
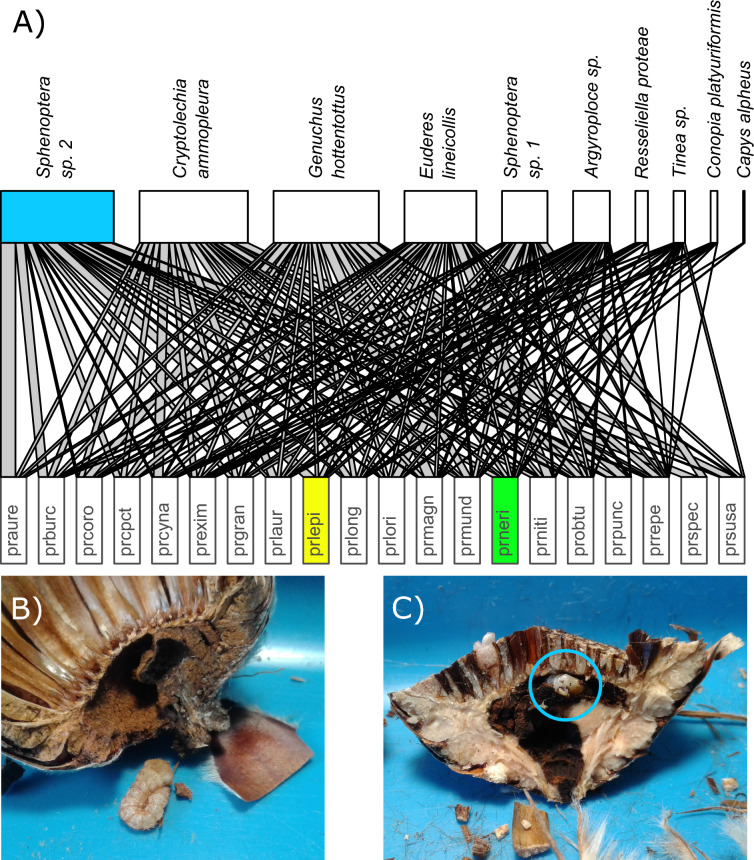
**A** Interaction network based on plant–herbivore interactions recorded in protea cones at 18 study sites in the Western Cape, South Africa. The upper bars represent herbivorous insect species and the lower bars represent *Protea* species (for plant species codes, see Table S3). The width of the upper bars indicates the relative number of observed interactions for each herbivorous insect species, and the link width indicates the relative interaction frequency between the respective *Protea* and its herbivorous insect species. The size of the lower bars is standardized to represent 100% of the herbivore interactions with the respective *Protea* species. **B** A flatheaded borer (*Sphenoptera sp. 2*, family: Buprestidae, the third most frequent herbivorous insect species, blue in Fig. 1 A) taken from the cut open receptacle of *Protea lepidocarpodendron* (yellow in Fig. 1 A)*.* The larva destroyed the vascular tissue in the receptacle, which led to the opening of the cone and the release of all seeds. **C** The same flathead borer species inside its borehole (blue circle) in the receptacle of *Protea neriifolia* (green in Fig. 1 A). Picture copyrights by the authors

Overall, we recorded 984 interactions of ten herbivorous insect species in 1,173 cones of 20 *Protea* species (Fig. 1, Table S2). Coleoptera was the most abundant order and contributed to 68% of the total interactions, followed by Lepidoptera (31%) and Diptera (0.01%) (Table S2). The most abundant herbivorous insect species were the scarab beetle *Genuchus hottentottus* and the moth *Cryptolechia ammopleura* with 250 (25%) and 201 (20%) interactions, respectively. Herbivorous insect species were mostly widespread, as four species occurred at all 18 sites, and only three species were found at fewer than ten sites. Except for the rare species *Capys alpheus* and *Conopia platyuriformis*, all herbivores interacted with many different *Protea* species (Fig. 1). For example, *Genuchus hottentottus* interacted with six of the seven *Protea* species at the site with the largest resource availability, “held_2” (Table S1).

**Fig. 2:**
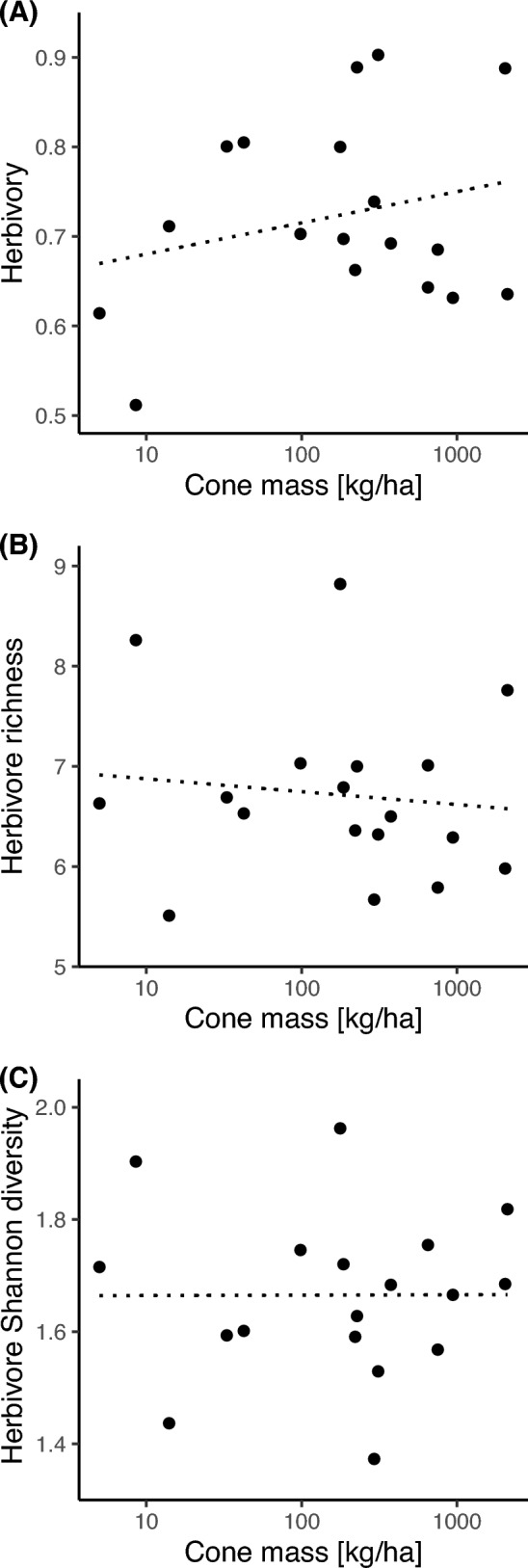
**A** Relationship of resource availability (cone mass [kg/ha]) with herbivory, **B** herbivore richness, and **C** herbivore Shannon diversity of 18 herbivorous insect communities in *Protea* cones in South African fynbos. Non-significant trend lines (*P* > 0.05) are indicated by dotted lines (see Table 1 for test statistics)

**Fig. 3:**
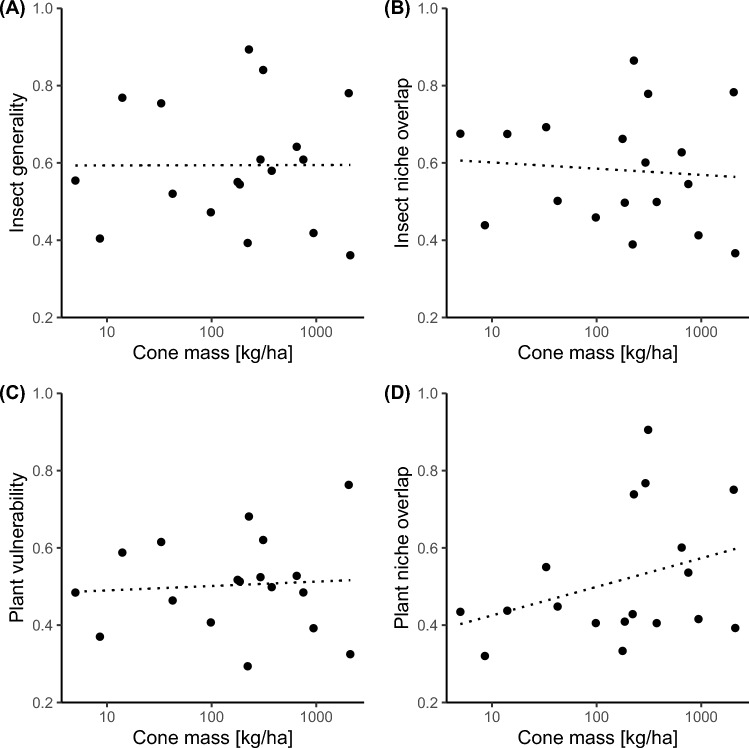
Relationship of resource availability with **A** insect generality, **B** insect niche overlap, **C** plant vulnerability, and **D** plant niche overlap of 18 herbivore communities in *Protea* cones in South African fynbos. Non-significant trend lines are indicated by dotted lines (see Table 2 for test statistics of multiple regressions). Insect generality was standardized by the number of *Protea* species per site, and plant vulnerability by the number of herbivore species per site

We did not find a relationship between community-level measures of herbivory and herbivore diversity and site-level resource availability or time since fire (Fig. 2; Table 1). Herbivory (i.e., the proportion of sampled cones attacked by herbivorous insects) was generally high (> 60% of infested cones at almost all sites), but it was unrelated to site-level resource availability and time since fire (Fig. 2A; Table 1A). Herbivore species richness ranged from 5.51 to 8.82 (mean = 6.719, SD = 0.86), and herbivore Shannon diversity ranged from 1.424 to 1.979 (mean = 1.665, SD = 0.15) per site. Despite the broad ranges in site-level resource availability and time since fire, both herbivore richness and diversity did not vary with resource availability and time since fire (Fig. 2B, C; Table 1B, C). Site-level network metrics varied among study sites (Fig. 3). For instance, herbivores used on average between 36 and 89% of the available *Protea* species at a site (Fig. 3A). However, insect generality was unrelated to resource availability and time since fire (Fig. 3A; Table 2A). Similarly, plant vulnerability was variable, ranging between 0.29 and 0.76, but was unrelated to both resource availability and time since fire (Fig. 3C, Table 2C). Measures of niche overlap between herbivores and plants were not associated with resource availability and time since fire (Fig. 3, Table 2).

**Table 1:** Model summaries of the associations of resource availability (mean cone mass per site) and time since fire (i.e., years since the last fire) with herbivory (A), herbivore richness (B), and herbivore Shannon diversity (C) recorded in protea cones at 18 study sites in the Western Cape, South Africa

Fixed effects	Estimate	Std. error	*t* value	*P*
*Herbivory*				
Intercept	0.403	0.194	2.076	0.056
Cone mass	0.018	0.014	1.253	0.229
Time since fire	0.006	0.004	1.470	0.162
*Herbivore richness*				
Intercept	8.770	1.566	5.599	0.000
Cone mass	−0.081	0.114	−0.713	0.487
Time since fire	−0.063	0.035	−1.826	0.088
*Herbivore Shannon diversity*				
Intercept	1.870	0.277	6.763	0.000
Cone mass	−0.004	0.020	−0.176	0.863
Time since fire	−0.010	0.006	−1.561	0.139

Shown are the main effects of resource availability and time since fire. Values represent model estimates along with their standard error, *t* value, and *p *value. Degrees of freedom = 15; sample size = 18 study sites. All response variables were rarefied to the minimum number of sampled cones per site (*n* = 36 cones)

**Table 2:** Model summaries of the associations of resource availability (cone mass in kg/ha) and time since fire (i.e., years since the last fire) with insect generality (A), insect niche overlap (B), plant vulnerability (C), and plant niche overlap (D) recorded in protea cones at 18 study sites in the Western Cape, South Africa

Fixed effects	Estimate	Std. error	*t* value	*p*
*Insect generality*				
Intercept	0.419	0.308	1.358	0.194
Cone mass	0.003	0.022	0.152	0.881
Time since fire	0.008	0.007	1.159	0.265
*Insect niche overlap*				
Intercept	0.559	0.290	1.925	0.073
Cone mass	−0.005	0.021	−0.239	0.815
Time since fire	0.005	0.006	0.763	0.457
*Plant vulnerability*				
Intercept	0.356	0.240	1.481	0.159
Cone mass	0.007	0.017	0.377	0.712
Time since fire	0.004	0.005	0.764	0.457
*Plant niche overlap*				
Intercept	−0.062	0.305	−0.204	0.841
Cone mass	0.036	0.022	1.612	0.128
Time since fire	0.009	0.007	1.299	0.214

Shown are the main effects of resource availability and time since fire. Values represent model estimates along with their standard error, *t* value, and *p *value. Degrees of freedom = 15; sample size = 18 study sites. All response variables were rarefied to the minimum number of sampled cones per site (*n* = 36 cones). Insect generality was standardized by the number of *Protea* species per site, and plant vulnerability by the number of herbivore species per site

## Discussion

We studied how endophagous insect herbivore communities and plant–herbivore interactions vary in response to resource availability and time since fire. Despite the broad environmental gradients studied, community-level measures of herbivory and plant–herbivore interactions were unrelated to the studied environmental gradients, suggesting that the mechanisms structuring the interactions in this plant–herbivore system are independent of the environmental context. Although the studied insect herbivores are highly specialized on the genus *Protea*, the plant–herbivore interactions studied here are characterized by a high degree of generalization, which may explain why herbivore diversity and specialization did not respond to site-level resource availability.

### Herbivory and herbivore diversity

*Protea* species were exposed to constant herbivore pressure across the studied gradients in resource availability and time since the last fire. The cones of each *Protea* species at all sites were exploited by almost all present herbivore species, even though we rarely found more than one herbivore species and individual per cone, suggesting high intra- and interspecific competition among herbivores. The high generalization in this plant–herbivore system was also corroborated by the network analysis, showing that the insect herbivores could use most of the available *Protea* species at a site.

One reason for the weak effects on herbivory and herbivore diversity could lie in the age structure of our study sites (range: 6–25 years). Slow-maturing overstorey *Protea* species may take up to 14 years to reach maturity (Geerts 2021). However, on our sites, many protea plants already reproduced after several years, suggesting that fynbos plant communities can recover quickly after a fire (Hope et al. 2012). Plant community dynamics likely contribute to the recovery of herbivorous insect populations and can contribute to a quick recovery of insect herbivore populations (Kim and Holt 2012; Gerisch 2014; Bosc and Pauw 2020). For example, a comparative study between burned and control plots in fynbos showed that the composition of foliage-feeding herbivorous insects already converged toward pre-fire conditions within 3 years post-fire, following a similar trajectory as plant species composition (Pryke and Samways 2012). Unlike other insect groups, insect herbivores are more likely to be affected by the indirect effects of fire, i.e., through changes in habitat structure (e.g., loss of shelter and reproduction sites) and plant resource availability, than by the direct effects of fire (Kim and Holt 2012). In line with that, our findings suggest that the insect herbivore communities that feed inside protea cones recolonize a site as soon as the *Protea* species flower for the first time. Since all studied herbivores have flight capacity and the extent of many fires is patchy, it can be assumed that the adult insect herbivores can navigate quickly to their host plants after settling in a disturbed habitat. Future studies, including recently burned sites, may test whether these quick recovery rates are primarily due to the local persistence of herbivore species at a site or to recolonization events in the first years after fire from neighboring, unburned sites.

Herbivory and herbivore diversity also showed little variation with changes in resource availability. In contrast to our findings, the resource concentration hypothesis states that greater immigration of herbivores to sites with high resource density will lead to higher herbivore density (Janzen 1970; Root 1973; Jactel et al. 2021), whereas the resource dilution hypothesis states that a resource dilution effect may lead to lower herbivore density at high resource availability (Otway et al. 2005). Our findings support neither of these two hypotheses. Our results rather suggest that the diversity of insect herbivores in Protea cones is largely unaffected by resource availability and that the proportion of cones containing herbivores remained constant along the resource gradient. This is in line with a study by Walter et al. (2022) who reported constant levels of predation in Protea communities, suggesting that herbivorous insect abundance changes proportionally with their available plant resources. Studies from other plant–herbivore systems found less support for synchronized community dynamics of plants and herbivores (Calixto et al. 2023). The coupled dynamics of plant and herbivore communities in our study system may be the result of a co-adaptation between the genus *Protea* and their herbivorous insects, due to similar strategies of plants and herbivores to cope with frequent fires (Procheş and Cowling 2006) and strong reciprocal selective pressures on Protea plants and their endophagous seed predators.

Across the studied gradients, herbivory was high as the ratio of infested cones was above 60% at almost all sites (Roets et al. 2006; Nottebrock et al. 2013; Sasa and Samways 2015). Even though herbivores can feed on many different *Protea* species, the larvae show different feeding strategies within a protea cone and arrive at different stages of cone maturity (Coetzee and Giliomee 1987a). For example, *Tinea sp.* (Tineidae) occurs only in mature cones and tends to be more frequent in cones with thin bracts (Coetzee and Giliomee 1987a; Neu et al. 2023), as thick and woody cone bracts shield the receptacle from some insect herbivores. In comparison, the most common species *Genuchus hottentottus* (Scarabaeidae) enters and leaves the cone via the top (Coetzee and Giliomee 1987a). Given the high herbivory rates on all *Protea* species, *Protea* species appear to be vulnerable to different types of insect herbivores. However, unpredictable seed set in cones may ensure successful reproduction despite high herbivory rates (Esler and Cowling 1990; Wright 1994; Wright and Samways 1999). In addition to high rates of insect herbivory, most protea plants simultaneously suffer high rates of predation from mammals that usually harvest entire cones (Botha and Pauw 2017). Consequently, *Protea* species have to invest heavily in reproduction to compensate for the generally high herbivore pressure in this system (Cooksley et al. 2023).

### Herbivore diversity and specialization

Contrary to our second hypothesis, we found that site-level resource availability was unrelated to herbivore specialization on plants. Since herbivory rates were also independent of resource availability, it is likely that the mechanisms structuring these interactions were independent of the environmental context. In fact, almost all herbivore species were able to feed on all *Protea* species. Other studies have also shown that herbivorous insects in protea communities are highly generalized, as most herbivore species occurred in the cones of most *Protea* species (Wright and Samways 1999; Neu et al. 2023). This suggests that there are few physical or physiological barriers to enable exploitation of host plants (Roets et al. 2006; Nottebrock et al. 2017b; Neu et al. 2023), which is also corroborated by the generally high niche overlap among insect species. Nevertheless, the interactions may be affected by competitive interactions between herbivores. Neu et al. (2023) suggest that competition among herbivores leads to energetic trait matching, so that the larger and therefore competitively superior herbivores dominate the most rewarding plant resources. In our study, we only found a single herbivorous larva in most cones, indicating that intra-guild predation and competition for limited resources may only allow for a single larva to survive per cone. Consequently, it seems likely that herbivorous insect species can use fewer *Protea* host species if competition is high. Such changes in competitive interactions between insect herbivores would be in line with classic competition theory, which postulates that competition increases when more individuals compete for limited resources (Levins 1968). However, in this study, we could not detect differences in overall herbivory rates, suggesting that the intensity of competition was independent of site-level resource availability, leading to similar rates of specialization along the environmental gradient.

Contrary to our findings, Bosc and Pauw (2020) showed that specialization of herbivorous insect species increases with time since disturbance in fynbos. This was driven by changes in the herbivore community in the first years after disturbance and the replacement of free-feeding generalists (mostly associated with grasses) by more specialized herbivore species associated with specific shrub and tree species. Similarly, Redmond et al. (2019) found that the vulnerability of plants to herbivores was highest in young successional stages in tropical montane forests. These studies suggest that disturbance may have distinct effects on free-feeding herbivore species that primarily depend on leaf resources compared to endophagous larvae of herbivores. Our findings from the protea system suggest that endophagous herbivores can quickly colonize a site once seed resources are available. Because the studied herbivore species are widespread in the study area (e.g., Roets et al. 2006), it is likely that these species have a high recolonization capacity from neighboring, unburned sites. Generally, we expect that our findings can be generalized to other endophagous insect species, whereas other feeding types of herbivores may be coupled to a lesser extent to their host plant communities.

## Conclusion

Our study provides new insights into the interactions between closely associated plant and herbivore species. We found surprisingly weak effects of plant resource availability and time since fire on herbivory and plant–herbivore interactions, suggesting a strong coupling of plant and herbivore communities. Coupled community dynamics of plants and insect herbivores may explain why plant–herbivore networks are similarly structured throughout the broad environmental gradients in South African fynbos.

## Supplementary Information

Below is the link to the electronic supplementary material.

Supplementary file1 (DOCX 29 KB)

## Data Availability

The raw data that support the findings of this study are openly available at Dryad Digital Repository: 10.5061/dryad.nvx0k6dwm.

## Code availability

Not applicable as the analysis is based on standard analytical procedures.
