# Supplementary material for: Constant herbivory rates and plant–herbivore interactions along a resource availability gradient in South African fynbos
Source: Oecologia. 2025 Oct 8;207(11):170. doi: 10.1007/s00442-025-05793-2 (PMC12507943; doi:10.1007/s00442-025-05793-2)
Supplement: Supplementary file 1 — Supplementary file1 (DOCX 29 KB) [file 442_2025_5793_MOESM1_ESM.docx]

## Supplement material for

# Constant herbivory rates and plant-herbivore interactions along a resource availability gradient in South African fynbos

Alexander Neu^1,2^, Huw Cooksley^3^, Karen J. Esler^4^, Anton Pauw^5^, Francois Roets^4^, Frank M. Schurr^3^ & Matthias Schleuning^1^

^1^Senckenberg Biodiversity and Climate Research Centre (SBiK-F), Frankfurt am Main, D

^2^Institute for Ecology, Evolution and Diversity, Goethe University Frankfurt, Frankfurt am Main, Germany, D

^3^Institute of Landscape and Plant Ecology, University of Hohenheim, D

^4^Department of Conservation Ecology and Entomology, Stellenbosch University, ZA

^5^Department of Botany and Zoology, Stellenbosch University, ZA

Table S1. All 18 study sites in the Western Cape, South Africa, their latitude and longitude, the predicted mean site-level protea cone mass [g], and the date of the last occurring fire. We chose the fifteenth of a month when the exact day of the fire was not known. Given are the number of recorded interactions, the number of herbivorous insect species (H/site), the number of sampled *Protea* species (P/site), and the total number of sampled cones (Cones/site).

| **Site** | **Lat** | **Long** | **Cone mass [g/ha]** | **Last fire** | **Interactions** | **H/site** | **P/site** | **Cones/site** |
| --- | --- | --- | --- | --- | --- | --- | --- | --- |
| cool_1 | -34.2 | 18.8 | 752881 | 1-May-89 | 62 | 7 | 3 | 84 |
| cool_2 | -34.2 | 18.8 | 311040 | 1-May-89 | 54 | 7 | 2 | 51 |
| fern_1 | -34.4 | 19.2 | 2044662 | 15-Oct-00 | 54 | 6 | 3 | 53 |
| fern_2 | -34.4 | 19.3 | 293230 | 15-Nov-01 | 64 | 6 | 4 | 72 |
| flow_1 | -34.6 | 19.5 | 941782 | 15-Feb-06 | 72 | 7 | 6 | 103 |
| groo_1 | -34.5 | 19.5 | 227302 | 15-Feb-06 | 36 | 7 | 2 | 36 |
| groo_4 | -34.5 | 19.5 | 8562 | 15-Feb-06 | 27 | 9 | 4 | 45 |
| held_2 | -34.1 | 18.9 | 2115268 | 16-Apr-11 | 95 | 9 | 7 | 114 |
| heun_1 | -34.3 | 19.1 | 186032 | 15-Nov-99 | 35 | 7 | 3 | 43 |
| jona_1 | -33.9 | 19.5 | 177202 | 15-Dec-00 | 39 | 9 | 3 | 40 |
| jona_2 | -33.9 | 19.5 | 375435 | 15-Dec-00 | 53 | 7 | 3 | 59 |
| jona_3 | -34.0 | 19.5 | 98300 | 15-Apr-92 | 54 | 8 | 5 | 67 |
| jona_4 | -34.0 | 19.5 | 42421 | 15-Dec-00 | 66 | 7 | 4 | 74 |
| lowy_1 | -34.1 | 18.9 | 650767 | 19-Jan-06 | 55 | 8 | 3 | 67 |
| palm_1 | -34.0 | 19.0 | 221272 | 25-May-98 | 84 | 9 | 6 | 112 |
| roch_1 | -33.9 | 19.2 | 14016 | 15-Feb-99 | 45 | 6 | 2 | 56 |
| roch_2 | -33.9 | 19.2 | 32964 | 15-Feb-99 | 51 | 7 | 2 | 50 |
| roch_3 | -33.9 | 19.2 | 4990 | 15-Feb-99 | 38 | 7 | 3 | 47 |

Table S2: The ten herbivorous insect species observed in protea cones on all 18 sites in the Western Cape, South Africa and their total number of interactions recorded across all study sites. Information on key traits of these species is given in Neu et al. (2023).

| **Herbivorous insect species** | **Family** | **Order** | **Interactions** |
| --- | --- | --- | --- |
| *Argyroploce sp.* | Tortricidae | Lepidoptera | 74 |
| *Capys alpheus* | Lycaenidae | Lepidoptera | 3 |
| *Conopia platyuriformis* | Sesiidae | Lepidoptera | 11 |
| *Cryptolechia ammopleura* | Depressariidae | Lepidoptera | 201 |
| *Euderes lineicollis* | Curculionidae | Coleoptera | 133 |
| *Genuchus hottentottus* | Scarabaeidae | Coleoptera | 250 |
| *Resseliella proteae* | Cecidomyiidae | Diptera | 12 |
| *Sphenoptera sp. 1* | Buprestidae | Coleoptera | 97 |
| *Sphenoptera sp. 2* | Buprestidae | Coleoptera | 187 |
| *Tinea sp.* | Tineidae | Lepidoptera | 16 |

Table S3. The 20 overstorey *Protea* species and their species codes in Fig. 1. Information on key traits of these species is given in Neu et al. (2023).

| **Species** | **Species code** |
| --- | --- |
| *Protea aurea* | praure |
| *Protea burchellii* | prburc |
| *Protea compacta* | prcpct |
| *Protea coronata* | prcoro |
| *Protea cynaroides* | prcyna |
| *Protea eximia* | prexim |
| *Protea grandiceps* | prgran |
| *Protea laurifolia* | prlaur |
| *Protea lepidocarpodendron* | prlepi |
| *Protea longifolia* | prlong |
| *Protea lorifolia* | prlori |
| *Protea magnifica* | prmagn |
| *Protea mundii* | prmund |
| *Protea neriifolia* | prneri |
| *Protea nitida* | prniti |
| *Protea obtusifolia* | probtu |
| *Protea punctata* | prpunc |
| *Protea repens* | prrepe |
| *Protea speciosa* | prspec |
| *Protea susannae* | prsusa |
